# Prevalence of Antibiotic Resistance Genes in Air-Conditioning Systems in Hospitals, Farms, and Residences

**DOI:** 10.3390/ijerph16050683

**Published:** 2019-02-26

**Authors:** Yaying Li, Hongkai Liao, Huaiying Yao

**Affiliations:** 1 Key Laboratory of Urban Environment and Health, Institute of Urban Environment, Chinese Academy of Sciences, Xiamen 361021, China; yyli@iue.ac.cn (Y.L.); hkliao@iue.ac.cn (H.L.); 2 Ningbo Key Laboratory of Urban Environmental Processes and Pollution Control, Ningbo Urban Environment Observation and Research Station—NUEORS, Institute of Urban Environment, Chinese Academy of Sciences, Ningbo 315800, China; 3 Research Center for Environmental Ecology and Engineering, School of Environmental Ecology and Biological Engineering, Wuhan Institute of Technology, Wuhan 430073, China

**Keywords:** antibiotic resistance genes, air-conditioning system, human health, indoor environment

## Abstract

High-throughput quantitative PCR combined with Illumina sequencing and network analysis were used to characterize the antibiotic resistance gene (ARG) profiles in air-conditioning filters from different environments. In total, 177 ARGs comprising 10 ARG types were determined. The detectable numbers and the relative abundance of ARGs in hospitals and farms were significantly higher than those in city and village residences. Compared to hospitals, farms had a higher level of tetracycline, multidrug, integrase, and macrolide–lincosamide–streptogramin (MLS) B resistance genes but a lower level of beta-lactam resistance genes. The *bl3_cpha* gene was the most abundant resistance gene subtype in hospital samples with an abundance of 2.01 × 10^−4^ copies/16S rRNA, while a level of only 5.08 × 10^−12^ copies/16S rRNA was observed in farm samples. There was no significant difference in bacterial diversity among the hospitals, farms, and residences, and Proteobacteria was the most abundant phylum. Network analysis revealed that Proteobacteria and Actinobacteria were possible hosts of the beta-lactam, MLSB, aminoglycoside, multidrug, sulfonamide, and tetracycline resistance genes. The results demonstrate that ARGs exist in indoor environments and that farms and hospitals are important sources. This study provides a useful reference for understanding the distribution patterns and risk management of ARGs in indoor environments.

## 1. Introduction

Medicine has been revolutionized by antibiotics throughout the 20th century, due to their ability to prevent and treat bacterial infections. However, the overuse and misuse of antibiotics prompted bacteria to develop resistance. Our capacity for treating infectious diseases is challenged by the increase in antibiotic-resistant bacteria (ARB) and antibiotic resistance genes (ARGs) [1]. Indeed, the World Health Organization classifies antimicrobial resistance as a “serious threat” to global public health [2]. 

This problem is especially prevalent in China because China is one of the largest producers and consumers of antibiotics in the world. More than 90 million tons of antibiotics are consumed annually in China, with approximately 48% procured for human use and 52% for agriculture [3]. The prescription of antibiotics accounts for about 10% of all drugs prescribed by hospitals in developed countries, but it exceeds 50% in China [4]. Cephalexin, amoxicillin, tetracycline, ofloxacin, and norfloxacin were the most frequently used antibiotics [3]. Antibiotics are usually used to control the disease and stimulate growth of animals in modern agriculture [5]. Amoxicillin, florfenicol, penicillin, lincomycin, and enrofloxacin were the five most frequently used antibiotics in the livestock industry, and the annual usage in China has exceeded 4000 tons [3,6]. Generally, antibiotics are incompletely absorbed in mammalian bodies and, hence, are excreted and dispersed into the environment. This aggravates the dissemination of ARB and ARGs in the environment. Therefore, hospital and animal husbandry are the primary contributors of antibiotic and ARG dissemination into receiving environments through waste disposal processes, municipal wastewater treatment, and application of manure to land.

Numerous studies have addressed the detection of antibiotics and ARGs in different environmental conditions, including air, water, sediment, sludge, and soil [7,8,9,10,11,12,13]. High-throughput quantitative PCR (HT-qPCR) combined with Illumina sequencing has been developed to provide a comprehensive insight into the profile of an extensive number of ARGs in different environmental samples [14,15,16,17]. Su et al. [18] used this method to characterize the geographical and seasonal distributions of ARGs and their potential hosts from 116 urban sewage samples. A total of 381 ARGs were determined and widely shared in all samples. The results demonstrated that human activity was the main driver of antibiotic resistance and its distribution. 

In recent times, humans spend the majority of time (almost 90%) indoors. There has been a growing concern over the health effects associated with indoor air quality since airborne diseases, including influenza [19], tuberculosis [20], the common cold [21], and legionellosis [22], exist in indoor air. Several studies have documented that humans who are exposed daily to these airborne particles can induce allergic and chronic inflammatory responses [23]. While current research in indoor air quality has mainly focused on pathogenic microorganisms, the occurrence and abundance of antibiotics and ARGs in indoor environments is still unclear.

Air-conditioning (AC) aims to achieve a more comfortable interior environment, particularly for humans and animals. However, AC systems allow microbial organisms to proliferate and infectious contaminants to accumulate through the collection of moisture and air exchange [24]. Extensive epidemiologic research has identified relationships between AC systems and indoor air quality [25]. In this study, 12 samples were collected from hospitals, farms, city residences, and village residences. HT-qPCR and Illumina sequencing of bacterial 16S rRNA gene were used (1) to comprehensively characterize the ARG profiles in air-conditioning filters from different sites; (2) to compare ARG profiles in different samples and locations, i.e., hospitals and farms; and (3) to reveal the correlations among the ARGs and bacteria in situ.

## 2. Materials and Methods

### 2.1. Site Selection and Sample Collection

A total of 12 samples were collected from four types of environment in Ningbo city (China) in October 2017. These environments included (1) hospitals (general hospital, medical hospital, surgical hospital); (2) farms (chicken farm, duck farm, swine farm); (3) city residences (3 samples); and (4) village residences (3 samples). Air-conditioning units had been running for the summer, and the air-conditioning filter had not been cleaned for 4 months. The dust in the air-conditioning filter was collected, sieved through a 2.0 mm mesh, and then stored at −20 °C for DNA extraction. 

### 2.2. DNA Extraction and Sequencing

The biomass from dust samples was too low to obtain sufficient DNA for HT-qPCR and Illumina sequencing analysis using commercial kits. Therefore, DNA was extracted from 2 g dust samples by previously described freeze-grinding and SDS lysis-based methods [26]. DNA was purified by electrophoresis on a low melting agarose gel, followed by phenol extraction according to Zhou et al. [27,28]. A NanoDrop spectrophotometer (ND-1000, Thermo Fisher Scientific, Waltham, MA, USA) was applied to assess DNA concentration and quality. All DNA samples were stored at −20 °C for later use.

In order to determine the diversity of microbial communities, the hypervariable V4 region of the 16S rRNA gene was amplified using the primer pair 515F (5’-GTGCCAGCMGCCGCGG-3’) and the reverse primer 907R (5’-CCGTCAATTCMTTTRAGTTT-3’) [29]. Each PCR amplification, using a 3 × 50 μL reaction system, was set up under the following program: 95 °C for 5 min; 30 cycles of 30 s at 95 °C, 30 s at 58 °C, 30 s at 72 °C; and a final extension step of 10 min at 72 °C. The PCR products were purified using the Universal DNA Purification kit (Tiangen, Beijing, China) and then quantified using the NanoDrop spectrophotometer (ND-1000, Thermo Fisher Scientific, Waltham, MA, USA). Pre-mixed samples were sent for sequencing at Novogene (Beijing, China) on an Illumina MiSeq PE300 platform.

### 2.3. Phylotype Analysis

All Illumina sequencing data were processed using the Quantitative Insights into Microbial Ecology (QIIME 1.9.1) platform as previously described [30]. Sequences were sorted and assigned by matching the unique barcode for each sample exactly. Low-quality reads, such as primer mismatches, ambiguous bases, and errors in barcodes were filtered out. Chimeric and noisy sequences were also removed. Qualified sequence reads were clustered into operational taxonomic units (OTUs) at 97% identity [31]. The most abundant OTU was conducted using a Ribosomal Database Project (RDP) Classifier at an 80% confidence threshold [32]. Rarefaction analysis was applied to determine Chao1 diversity, phylogenetic diversity, and observed species metrics at different sequencing depth. The reads were deposited into the NCBI short-reads archive database (accession number: SRP158950).

### 2.4. ARGs Determination

The WaferGen SmartChip Real-Time PCR system (WaferGen Inc., Fremont, CA, USA) with 296 validated primer sets was applied (Table S1). To determine the relative abundance of ARGs (copies/16S rRNA gene) as previously described [33], we targeted 285 ARGs from all major classes of antibiotics. Except for the 285 ARGs, the smart-chip system also included eight transposases, one class 1 integron-integrase gene (intI1), one clinical class 1 integron-integrase gene (cintI1), and one 16S rRNA gene. qPCR reactions were performed according to a previously published study by An et al. [34] with a 100 nL reaction system. Melting curve analysis was performed after amplification. 

### 2.5. Analysis of ARGs

SmartChip qPCR software (V 2.7.0.1, WaferGen Inc., Mountain View, CA, USA) was applied to analyze HT-qPCR data. Multipeak reactions and reactions with amplification efficiency out of the acceptable range (0.9–1.1) were removed [33,35,36]. In this study, a cycle threshold (Ct) value <31 was regarded as positive. Moreover, samples without three positive technical replicates were discarded. The relative abundance (copies/16S rRNA gene) of ARGs were calculated according to Looft et al. [37] (see Equation (1)).

Gene copy number = 10^(31−Ct)/(10/3)^ (1)

### 2.6. Statistical and Network Analysis

Excel 2010 (Microsoft Office 2010, Microsoft, Redmond, WA, USA) was applied to determine the averages and standard deviations. One-way analysis of variation (ANOVA) and principal component analysis (PCA) were performed using SPSS version 18.0 (IBM, Armonk, NY, USA). For testing of statistically significant differences, a significance level of 0.05 was used. Heatmap plotting was performed in R 3.5.0 with pheatmap package. To investigate co-occurrence patterns of microbial communities and ARGs, correlation matrices were constructed by calculating all possible pairwise Spearman’s rank correlations between ARG subtypes and bacterial genera in R environment using the “psych” package. A correlation between any two items was considered to be a robust correlation if the Spearman’s correlation coefficient (ρ) was >0.7 and the *p* value was <0.01. Network visualization was conducted using the interactive platform Gephi 0.9.1 software (https://gephi.org/).

## 3. Results

### 3.1. Occurrence and Abundance of ARGs

**Figure 1:**
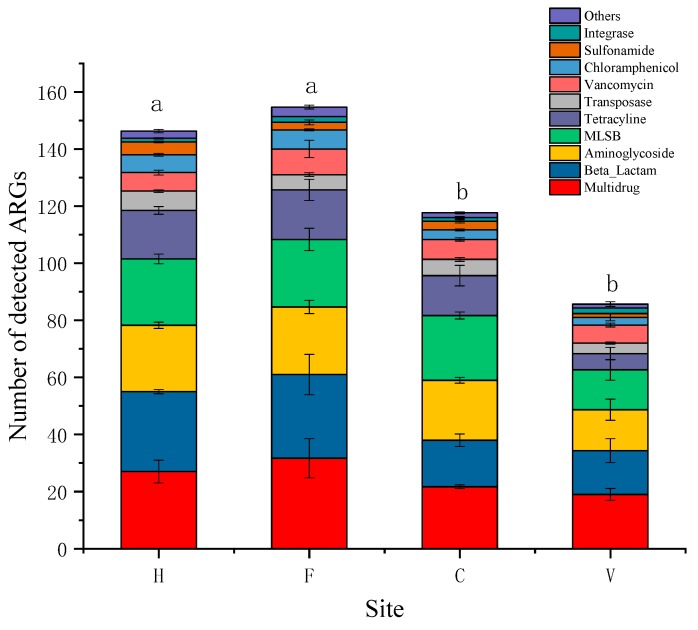
The number of detected antibiotic resistance gene (ARG) types in air-conditioning filters. H: hospital; F: farm; C: city residence; V: village residence. MLSB: macrolide–lincosamide–streptogramin B resistance. Error bars indicate standard errors of three replicates, different lowercase letters indicate a significant difference (*p* < 0.05).

**Figure 2:**
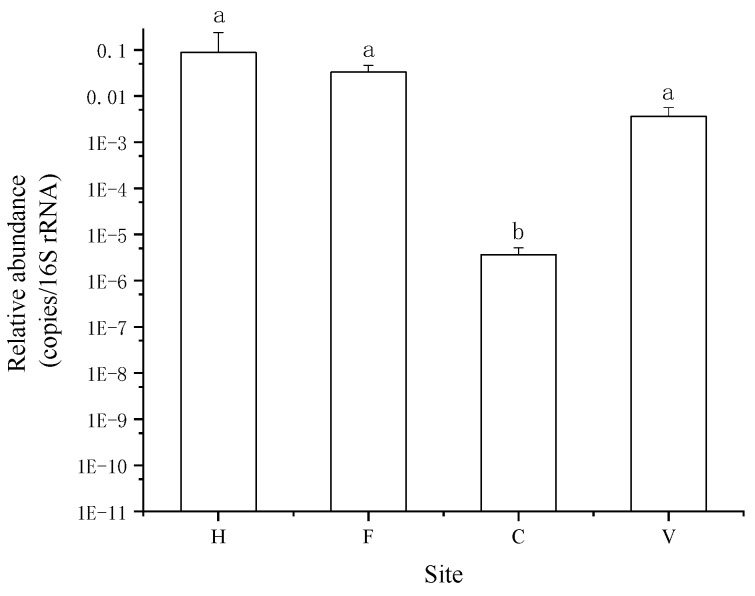
The relative copy number (copies/16S rRNA, relative abundance) of ARGs. H: hospital; F: farm; C: city residence; V: village residence. Error bars indicate standard errors of three replicates, different lowercase letters indicate a significant difference (*p* < 0.05).

In total, 177 ARGs were detected in all samples. Hospital and farm locations were major sources of antibiotics, which contained 146 and 154 ARGs, respectively. However, in city and village samples, ARGs were also detected, numbering 103 and 85, respectively. These were significantly lower than in hospital and farm samples. The composition of ARG types in the four treatments were similar; multidrug, beta-lactam, aminoglycoside, tetracycline, and macrolide–lincosamide–streptogramin B resistance (MLSB) were the five most dominant resistance gene types, comprising 78.7% to 81.3% of the total detected ARGs (Figure 1). The relative abundances of ARGs (copies/16S rRNA) in hospital, farm, city, and village locations were 8.76 × 10^−2^, 3.29 × 10^−2^, 3.64 × 10^−6^, and 3.29 × 10^−3^ copies/16S rRNA, respectively. The relative abundance of ARGs in city samples was the lowest, and significantly lower than in the other three locations (Figure 2). 

**Figure 3:**
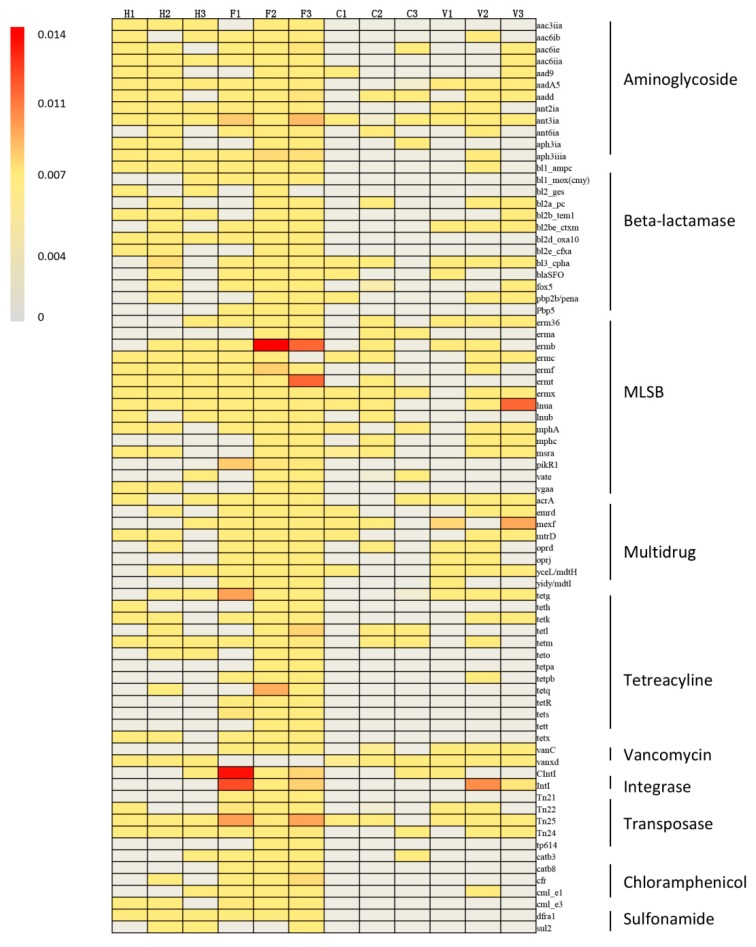
The heat map of detected ARGs. H: hospital; F: farm; C: city residence; V: village residence. MLSB: macrolide–lincosamide–streptogramin B resistance.

**Figure 4:**
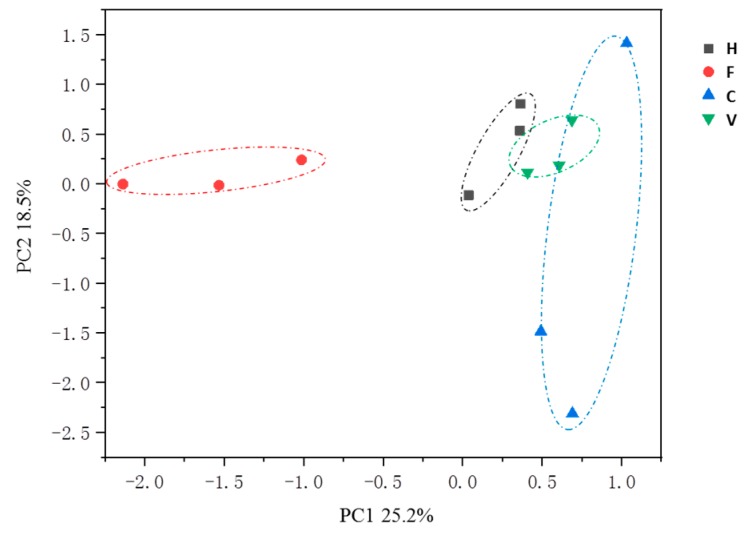
The ARG composition by principle component analysis (PCA). H: hospital; F: farm; C: city residence; V: village residence.

The top 79 most abundant ARGs were extracted and are shown in Figure 3. MLSB, beta-lactam, multidrug, integrase, and tetracycline resistance genes were the most abundant ARG types. All of these ARGs were detected in farm samples except for the *vanxd* gene, while *ermb* and *CIntI* were the two most abundant subtypes. Resistance gene *bl3_cpha* was the most abundant gene identified in hospital and village samples (6.03 × 10^−4^ and 8.79 × 10^−2^ copies/16S rRNA, respectively), while the number and relative abundance of ARGs was higher in hospital samples compared with village samples. The similarity of ARG compositions in all 12 samples was determined by PCA (Figure 4). The ARG profiles from farms significantly separated along PC1, indicating that the farms had different resistome profiles. No distinct difference was observed between hospital, city, and village samples. Factor loadings of individual resistance genes on PC1 confirmed that *ant3ia* (score −0.639), *qacEdelta1* (score −0.402), and *Tn25* (score −0.338) were relatively abundant in farm samples.

**Figure 5:**
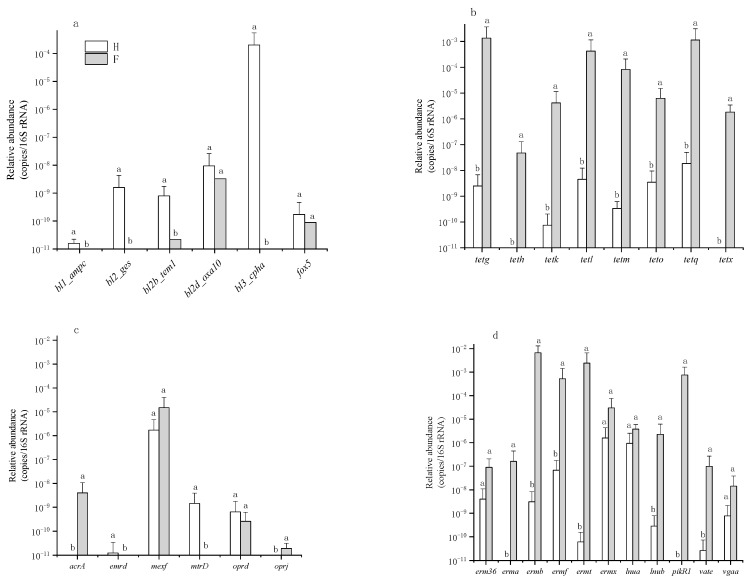
The relative abundance of (**a**) beta-lactam resistance genes, (**b**) tetracycline resistance genes, (**c**) multidrug resistance genes, and (**d**) MLSB resistance genes. H: hospital; F: farm. MLSB: macrolide–lincosamide–streptogramin B resistance. Error bars indicate standard errors of three replicates, different lowercase letters indicate a significant difference (*p* < 0.05).

The most frequently prescribed antibiotics in hospitals and farms were beta-lactam and tetracycline, respectively [38]. Therefore, the beta-lactam and tetracycline resistance genes were analyzed in both hospital and farm samples. Six beta-lactam resistance genes (*bl1_ampC*, *bl2_ges*, *bl2b_tem1*, *bl2d_oxa10*, *bl3_cpha*, *fox5*) and eight tetracycline resistance genes (*tetg*, *teth*, *tetk*, *tetl*, *tetm*, *teto*, *tetq*, *tetx*) were detected in both hospital and farm samples. Compared to farm samples, hospitals had a higher level of beta-lactam resistance genes and a lower level of tetracycline resistance genes (Figure 5a,b). The resistance gene *bl3_cpha* was the most abundant subtype in hospital samples with an abundance of 2.01 × 10^−4^ copies/16S rRNA, while in farm samples, the *bl3_cpha* gene abundance was only 5.08 × 10^−12^ copies/16S rRNA. The resistance genes *tetq* and *tetg* were the most abundant subtypes in farm samples with abundances of 1.14 × 10^−3^ and 1.36 × 10^−3^ copies/16S rRNA, respectively, while *tetq* and *tetg* gene abundances were only 1.84 × 10^−8^ and 2.49 × 10^−9^ copies/16S rRNA in hospital samples, respectively. Moreover, the relative abundance of multidrug and MLSB resistance genes were also higher in farm samples than in hospital samples (Figure 5c,d).

### 3.2. Characterization of Bacterial Community

**Figure 6:**
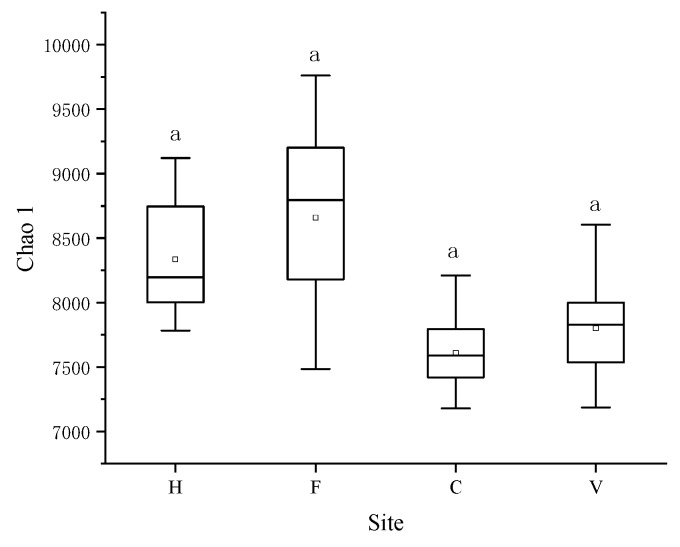
Rarefied Chao 1 index. H: hospital; F: farm; C: city residence; V: village residence. Error bars indicate standard errors of three replicates, different lowercase letters indicate a significant difference (*p* < 0.05).

**Figure 7:**
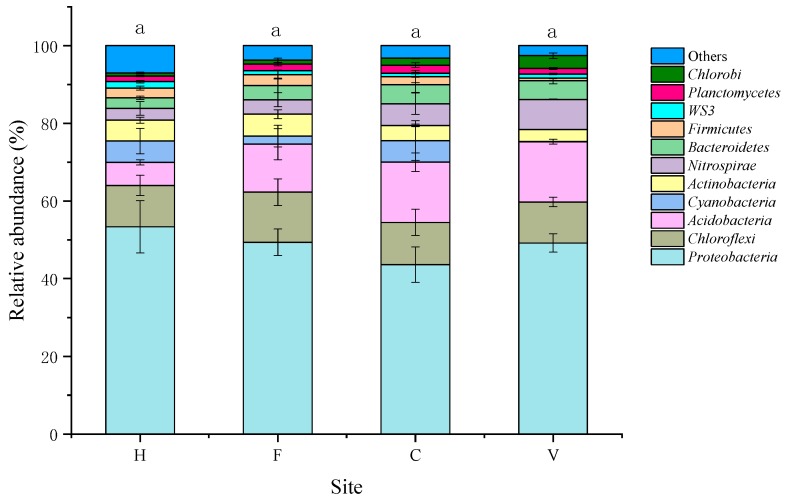
The abundance of dominant bacteria at the phylum level. H: hospital; F: farm; C: city residence; V: village residence. Error bars indicate standard errors of three replicates, different lowercase letters indicate a significant difference (*p* < 0.05).

In total, 661,301 high quality sequences (ranging from 27,303 to 88,263 sequences per sample, median = 53,302) were obtained from 12 samples, which were clustered into 25,311 OTUs at 97% similarity cutoff. There was no significant difference in bacterial diversity (chao1 index) among the four treatments (Figure 6). Proteobacteria, Chloroflexi, Acidobacteria, Actinobacteria, Nitrospirae, and Cyanobacteria were the six dominant phyla, accounting for 83.8% to 86.2% of the total abundance within the 12 samples (Figure 7). Proteobacteria were the most abundant phylum, and the relative abundances were 43.6% to 53.4% in the four treatments. Some pathogenic microorganisms, such as *Staphylococcus, Enterobacteriaceae, Pseudomonas,* and *Corynebacterium* were also detected.

### 3.3. Correlation of ARGs with Bacterial Community

**Figure 8:**
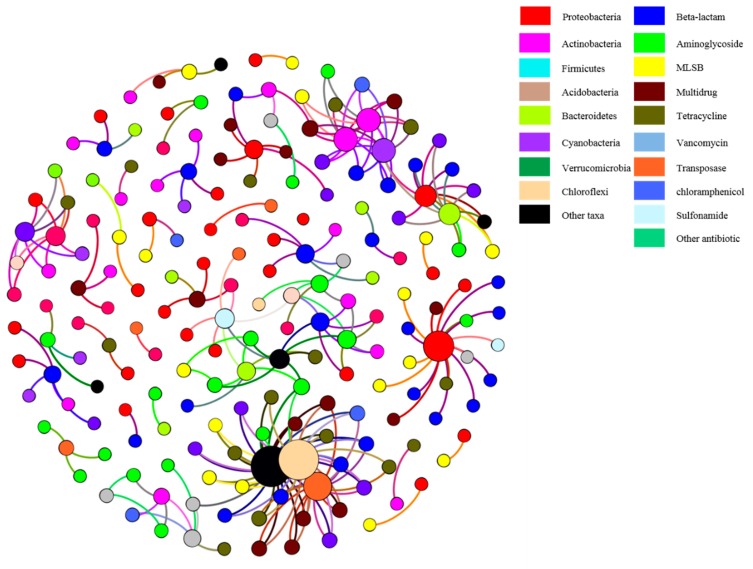
The co-occurrence patterns among ARGs and microbial taxa. The nodes were colored according to ARG types and genus. The size of each node is proportional to the number of connections, and the thickness of each connection between two nodes is proportional to the value of Spearman’s rank correlation coefficients ρ > 0.7. MLSB: macrolide–lincosamide–streptogramin B resistance.

Co-occurrence patterns between ARGs and bacterial taxa (at the genus level) were performed by network analysis. The correlation analysis showed strong (ρ > 0.7) and significant (*p* < 0.01) correlations between ARGs and bacterial assemblages. Proteobacteria, Actinobacteria, and Chloroflexi were the potential hosts of ARGs. Proteobacteria had non-random connections with beta-lactam, MLSB, aminoglycoside, multidrug, sulfonamide, and tetracycline resistance genes. Actinobacteria was a potential host of beta-lactam, aminoglycoside, multidrug, and tetracycline resistance genes. The multidrug ARGs were mainly associated with Proteobacteria and Actinobacteria (Figure 8). 

## 4. Discussion

In this study, we provide detailed information on ARG profiles in air-conditioning filters from hospitals, farms, and residences. Although the relative abundances of ARGs in AC filters were much lower (less than 1–5 orders of magnitude) than those of soil, sludge, and wastewater [18,34,39,40], 10 resistance gene types—including 177 subtypes—were determined, including somemultidrug resistance genes. Environmental ARGs can be transferred to both pathogenic and nonpathogenic microorganisms through the lateral transfer of genes [41,42], leading to drug resistance by inhalation of ARGs located within pathogens for susceptible populations [43]. These results suggest that the ARGs in AC units might be a possible public health threat through ventilation and respiration. 

Indoor air quality is of critical importance as humans now spend the majority of their time indoors. While current research in this field has mostly focused on the indoor microbiome, numerous studies have characterized the community composition, abundance, and specific sources of indoor microorganisms [44,45]. However, less is understood concerning the ARGs in indoor environments. Previous studies have shown that outdoor air could contribute to the variation in composition or abundance of microbes in air circulating indoors [46]. The diversity and abundance of ARGs in the particulate matter of severe smog were determined [47]. Similar to our results, a total of 205 airborne ARG subtypes were detected. Among the detectable ARGs, tetracycline, beta-lactam, aminoglycoside, chloramphenicol, MLSB, and multidrug resistance genes exhibited the highest abundance. However, integrase resistance genes were also abundant in farm samples in our study. Hu and colleagues found the levels of ARGs to be similar among airborne particulate matter, soil, and activated sludge, and these were all higher than observed in our study [47]. Shen et al. [48] suggested that automobile exhaust emissions, dust, and industrial gas could provide more attachment points for airborne microorganisms in smog. Moreover, particulate matter tends to be suspended in air, rather than deposited onto land [48].

Our results showed that the ARG abundances in farm and hospital locations were significantly higher than those in city and village residences. However, PCA revealed that ARG profiles from the farm samples were different compared to those of the hospital samples. With the exception of beta-lactam, the relative abundance of resistance genes for tetracycline, multidrug, integrase, and MLSB were significantly higher in the farm samples than in the hospital samples. Many types of antibiotics (especially tetracycline) are used in livestock industries, and intensive animal farming is considered to be a primary contributor to the increased environmental burden of ARGs. High-capacity qPCR arrays were used to study the drivers and abundant ARGs in three Chinese swine farms. A total of 149 unique ARGs were detected in all samples, which potentially conferred resistance to aminoglycosides, beta-lactam, tetracyclines, and MLSB [49]. Hong et al. [50] demonstrated that tetracycline resistance genes (*TetB*, *TetH*, *TetZ*, *TetO*, *TetQ*, and *TetW*) existed in high abundance, ranging from 9.55 × 10^2^ to 1.69 × 10^6^ copies per ng DNA extracted from the bioaerosols sampled from both pig and poultry farms. The relative abundances of the integron-integrase genes (*CIntI* and *IntI*) were considerably high in one farm sample in our study (Figure 3). *IntI* is frequently linked to ARGs and has penetrated into diverse pathogenic bacteria. Moreover, *IntI* is easily transmissible between diverse commensal and pathogenic bacteria associated with humans and animals, and could increase the risk of horizontal gene transfer [51]. Continued selective pressure by different antibiotics has resulted in multidrug resistance organisms and multidrug resistance genes [52]. A high abundance of multidrug resistance genes in farm samples were found in this study, which suggested that the microorganisms of farms could be resistant to multiple antibiotics. 

Beta-lactam is the most widely used antibiotic (occupies 65% of the total injectable antibiotics in hospital) due to its low toxicity and wide applicability [53,54]. Therefore, numerous studies have demonstrated that beta-lactam ARGs and beta-lactam-resistant bacteria were detected in sputum, urine, blood, wastewater, and air samples obtained from hospitals [55,56]. Rowe et al. [57] compared the abundances of beta-lactam ARGs in hospital and farm effluents. They found that two beta-lactam resistance genes were overexpressed in hospital effluent samples, but this overexpression was not observed in farm effluents. The high abundances of ARGs in effluents were mainly related to the heavy use of beta-lactam in hospitals.

Gao et al. [58] characterized the microbial community in five nosocomial bioaerosols. Similar to our results, they found that Proteobacteria represented the most prevalent microbes at almost 45% representation. *Staphylococcus saprophyticus*, *Corynebacterium minutissimum*, *Streptococcus pneumoniae*, *Escherichia coli*, *Arcobacter butzleri*, *Aeromonas veronii*, *Pseudomonas aeruginosa*, and *Bacillus cereus* were the most abundant pathogenic microorganisms. The non-random co-occurrence patterns between ARGs and the microbial community could indicate potential host information relating to ARGs, since specific microbial taxa appear to carry specific ARGs [8]. Therefore, network analysis is widely used to provide us with new insights into the ARGs and their role as potential hosts in complex environmental conditions [59]. Previous studies have identified some ARG hosts, such as *Lactobacillus*, which was considered to be a host to a number of ARGs, including gentamicin, tetracycline, and kanamycin [47]. Proteobacteria, Cyanobacteria, Bacteroidetes, and Verrucomicrobia were regarded as potential hosts of tetracycline resistance genes in coastal aquaculture [60]. In the present study, network analysis revealed that Proteobacteria and Actinobacteria were the possible hosts of resistance genes for beta-lactam, aminoglycoside, multidrug, and tetracycline. In our study, Proteobacteria and Actinobacteria were the first and fourth most abundant phyla, and this cannot be ruled out as a reason for the close association between the two phyla and ARGs.

## 5. Conclusions

The ARG profiles in air-conditioning filters from different environments were comprehensively characterized using HT-qPCR and Illumina sequencing. The detectable number and the relative abundances of ARGs in hospital and farm samples were significantly higher than in city and village samples, indicating that hospitals and farms were major sources of ARGs. With the exception of beta-lactam resistance genes, the relative abundance of resistance genes for tetracycline, multidrug, integrase, and MLSB were significantly higher in farm samples than in hospital samples. Our results suggested that the presence of ARGs was primarily linked to the heavy usage of antibiotics. Network analysis revealed that Proteobacteria and Actinobacteria were possible hosts of beta-lactam, MLSB, aminoglycoside, multidrug, sulfonamide, and tetracycline resistance genes. This study may help to better understand the general distribution patterns of ARGs in indoor environments, and more efforts should be devoted to exploring the relationship between ARGs and pathogenic microorganisms.

## Supplementary Materials

The following is available online at https://www.mdpi.com/1660-4601/16/5/683/s1. Table S1: Primer list of antibiotic resistance genes in this study.
