# Supplementary material for: Prevalence of Antibiotic Resistance Genes in Air-Conditioning Systems in Hospitals, Farms, and Residences
Source: Int J Environ Res Public Health. 2019 Feb 26;16(5):683. doi: 10.3390/ijerph16050683 (PMC6427721; doi:10.3390/ijerph16050683)
Supplement: Supplementary file 1 [file ijerph-16-00683-s001.pdf]

Supplementary Material: Prevalence of Antibiotic Resistance Genes in Air-Conditioning Systems in Hospitals, Farms, and Residences

The Supporting Information has 14 pages including 1 table.

Table S1: Primer list of antibiotic resistance genes in this study

Gene Name	Forward Primer	Reverse Primer	Classification
aac	CCCTGCGTTGTGGCTATGT	TTGGCCACGCCAATCC	Aminoglycoside
aac(6')I1	GACCGGATTAAGGCCGATG	CTTGCCTTGATATTCAGTTTTTATAACCA	Aminoglycoside
aac(6')-Ib(aka aacA4)-01	GTTTGAGAGGCAAGGTACCGTAA	GAATGCCTGGCGTGTTTGA	Aminoglycoside
aac(6')-Ib(aka aacA4)-02	CGTCGCCGAGCAACTTG	CGGTACCTTGCCTCTCAAACC	Aminoglycoside
aac(6')-Ib(aka aacA4)-03	AGAAGCACGCCCGACACTT	GCTCTCCATTCAGCATTGCA	Aminoglycoside
aac(6')-II	CGACCCGACTCCGAACAA	GCACGAATCCTGCCTTCTCA	Aminoglycoside
aac(6')-Iy	GCTTTGCGGATGCCTCAAT	GGAGAACAAAAATACCTTCAAGGAAA	Aminoglycoside
aacA/aphD	AGAGCCTTGGGAAGATGAAGTTT	TTGATCCATACCATAGACTATCTCATCA	Aminoglycoside
aacC	CGTCACTTATTCGATGCCCTTAC	GTCGGGCGCGGCATA	Aminoglycoside
aacC1	GGTCGTGAGTTCGGAGACGTA	GCAAGTTCCCGAGGTAATCG	Aminoglycoside
aacC2	ACGGCATTCTCGATTGCTTT	CCGAGCTTCACGTAAGCATTT	Aminoglycoside
aacC4	CGGCGTGGGACACGAT	AGGGAACCTTTGCCATCAACT	Aminoglycoside
aadA-01	GTTGTGCACGACGACATCATT	GGCTCGAAGATACCTGCAAGAA	Aminoglycoside
aadA-02	CGAGATTCTCCGCGCTGTA	GCTGCCATTCTCCAAATTGC	Aminoglycoside
aadA1	AGCTAAGCGCGAACTGCAAT	TGGCTCGAAGATACCTGCAA	Aminoglycoside
aadA-1-01	AAAAGCCCGAAGAGGAACTTG	CATCTTTCACAAAGATGTTGCTGTCT	Aminoglycoside
aadA-1-02	CGGAATTGAAAAAACTGATCGAA	ATACCGGCTGTCCGTCATTT	Aminoglycoside
aadA2-01	ACGGCTCCGCAGTGGAT	GGCCACAGTAACCAACAAATCA	Aminoglycoside
aadA2-02	CTTGTCGTGCATGACGACATC	TCGAAGATACCCGCAAGAATG	Aminoglycoside
aadA2-03	CAATGACATTCTTGCGGGTATC	GACCTACCAAGGCAACGCTATG	Aminoglycoside

aadA5-01	ATCACGATCTTGCGATTTTGCT	CTGCGGATGGGCCTAGAAG	Aminoglycoside
aadA5-02	GTTCTTGCTCTTGCTCGCATT	GATGCTCGGCAGGCAAAC	Aminoglycoside
aadA9-01	CGCGGCAAGCCTATCTTG	CAAATCAGCGACCGCAGACT	Aminoglycoside
aadA9-02	GGATGCACGCTTGGATGAA	CCTCTAGCGGCCGGAGTATT	Aminoglycoside
aadD	CCGACAACATTTCTACCATCCTT	ACCGAAGCGCTCGTCGTATA	Aminoglycoside
aadE	TACCTTATTGCCCTTGGAAGAGTTA	GGAACTATGTCCCTTTTAATTCTACAATCT	Aminoglycoside
aph	TTTCAGCAAGTGGATCATGTTAAAAT	CCAAGCTGTTTCCACTGTTTTTC	Aminoglycoside
aph(2')-Id-01	TGAGCAGTATCATAAGTTGAGTGAAAAG	GACAGAACAATCAATCTCTATGGAATG	Aminoglycoside
aph(2')-Id-02	TAAGGATATACCGACAGTTTTGGAAA	TTTAATCCCTCTTCATACCAATCCATA	Aminoglycoside
aph6ia	CCCATCCCATGTGTAAGGAAA	GCCACCGCTTCTGCTGTAC	Aminoglycoside
aphA1(aka kanR)	TGAACAAGTCTGGAAAGAAATGCA	CCTATTAATTTCCCCTCGTCAAAAA	Aminoglycoside
spcN-01	AAAAGTTCGATGAAACACGCCTAT	TCCAGTGGTAGTCCCCGAATC	Aminoglycoside
spcN-02	CAGAATCTTCCTGAAAAGTTTGATGAA	CGCAGACACGCCGAATC	Aminoglycoside
tr	AATGAGTTTTGGAGTGTCTCAACGTA	AATCAAAACCCCTATTAAAGCCAAT	Aminoglycoside
strA	CCGGTGGCATTTGAGAAAAA	GTGGCTCAACCTGCGAAAAG	Aminoglycoside
strB	GCTCGGTCGTGAGAACAATCT	CAATTTCGGTCGCCTGGTAGT	Aminoglycoside
ampC/blaDHA	TGGCCGCAGCAGAAAGA	CCGTTTTATGCACCCAGGAA	Beta_Lactam
ampC-01	TGGCGTATCGGGTCAATGT	CTCCACGGGCCAGTTGAG	Beta_Lactam
ampC-02	GCAGCACGCCCCGTAA	TGTACCCATGATGCGCGTACT	Beta_Lactam
ampC-04	TCCGGTGACGCGACAGA	CAGCACGCCGGTGAAAGT	Beta_Lactam
ampC-05	CTGTTCGAGCTGGGTTCTATAAGTAAA	CAGTATCTGGTCACCGGATCGT	Beta_Lactam
ampC-06	CCGCTCAAGCTGGACCATAC	CCATATCCTGCACGTTGGTTT	Beta_Lactam
ampC-07	CCGCCCAGAGCAAGGACTA	GCTCGACTTCACGCCGTAAG	Beta_Lactam
ampC-09	CAGCCGCTGATGAAAAAATATG	CAGCGAGCCCACTTCGA	Beta_Lactam
bla1	GCAAGTTGAAGCGAAAGAAAAGA	TACCAGTATCAATCGCATATACACCTAA	Beta_Lactam

bla-ACC-1	CACACAGCTGATGGCTTATCTAAAA	AATAAACGCGATGGGTTCCA	Beta_Lactam
blaCMY	CCGCGGCGAAATTAAGC	GCCACTGTTTGCCTGTCAGTT	Beta_Lactam
blaCMY2-01	AAAGCCTCAT GGGTGCATAAA	ATAGCTTTTGTTTGCCAGCATCA	Beta_Lactam
blaCMY2-02	GCGAGCAGCCTGAAGCA	CGGATGGGCTTGTCCTCTT	Beta_Lactam
blaCTX-M-01	GGAGGCGTGACGGCTTTT	TTCAGTGCGATCCAGACGAA	Beta_Lactam
blaCTX-M-02	GCCGCGGTGCTGAAGA	ATCGGATTATAGTTAACCAGGTCAGATTT	Beta_Lactam
blaCTX-M-03	CGATACCACCACGCCGTTA	GCATTGCCCAACGTCAGATT	Beta_Lactam
blaCTX-M-04	CTTGGCGTTGCGCTGAT	CGTTCATCGGCACGGTAGA	Beta_Lactam
blaCTX-M-05	GCGATAACGTGGCGATGAAT	GTCGAGACGGAACGTTTCGT	Beta_Lactam
blaCTX-M-06	CACAGTTGGTGACGTGGCTTAA	CTCCGCTGCCGGTTTTATC	Beta_Lactam
blaGES	GCAATGTGCTCAACGTTCAAG	GTGCCTGAGTCAATTCTTTCAAAG	Beta_Lactam
blaIMP-01	AACACGGTTTGGTGGTTCTTGTA	GCGCTCCACAAACCAATTG	Beta_Lactam
blaIMP-02	AAGGCAGCATTTCCTCTCATTTT	GGATAGATCGAGAATTAAGCCACTCT	Beta_Lactam
bla-L1	CACCGGGTTACCAGCTGAAG	GCGAAGCTGCGCTTGTAGTC	Beta_Lactam
blaMOX/blaCMY	CTATGTCAATGTGCCGAAGCA	GGCTTGTCCTCTTTCGAATAGC	Beta_Lactam
blaOCH	GGCGACTTGCGCCGTAT	TTTTCTGCTCGGCCATGAG	Beta_Lactam
blaOKP	GCCGCCATCACCATGAG	GGTGACGTTGTCACCGATCTG	Beta_Lactam
blaOXA1/blaOXA30	CGGATGGTTTGAAGGGTTTATTAT	TCTTGGCTTTTATGCTTGATGTTAA	Beta_Lactam
blaOXA10-01	CGCAATTATCGGCCTAGAAACT	TTGGCTTTCCGTCCCATTT	Beta_Lactam
blaOXA10-02	CGCAATTATCGGCCTAGAAACT	TTGGCTTTCCGTCCCATTT	Beta_Lactam
blaOXY	CGTTCAGGCGGCAGGTT	GCCGCGATATAAGATTTGAGAATT	Beta_Lactam
blaPAO	CGCCGTACAACCGGTGAT	GAAGTAATGCGGTTCTCCTTTCA	Beta_Lactam
blaPER	TGCTGGTTGCTGTTTTTGTGA	CCTGCGCAATGATAGCTTCAT	Beta_Lactam
blaPSE	TTGTGACCTATTCCCCTGTAATAGAA	TGCGAAGCACGCATCATC	Beta_Lactam
blaROB	GCAAAGGCATGACGATTGC	CGCGCTGTTGTCGCTAAA	Beta_Lactam

blaSFO	CCGCCGCCATCCAGTA	GGGCCGCCAAGATGCT	Beta_Lactam
blaSHV-01	TCCCATGATGAGCACCTTTAAA	TTCGTCACCGGCATCCA	Beta_Lactam
blaSHV-02	CTTTCCCATGATGAGCACCTTT	TCCTGCTGGCGATAGTGGAT	Beta_Lactam
blaTEM	AGCATCTTACGGATGGCATGA	TCCTCCGATCGTTGTCAGAAGT	Beta_Lactam
blaTLA	ACACTTTGCCATTGCTGTTTATGT	TGCAAATTTCGGCAATAATCTTT	Beta_Lactam
blaVEB	CCCGATGCAAAGCGTTATG	GAAAGATTCCCTTTATCTATCTCAGACAA	Beta_Lactam
blaVIM	GCACTTCTCGCGGAGATTG	CGACGGTGATGCGTACGTT	Beta_Lactam
blaZ	GGAGATAAAGTAACAAATCCAGTTAGATATGA	TGCTTAATTTTCCATTTGCGATAAG	Beta_Lactam
cepA	AGTTGCGCAGAACAGTCCTCTT	TCGTATCTTGCCCGTCGATAAT	Beta_Lactam
cfiA	GCAGCGTTGCTGGACACA	GTTCGGGATAAACGTGGTGACT	Beta_Lactam
cfxA	TCATTCCTCGTTCAAGTTTTCAGA	TGCAGCACCAAGAGGAGATGT	Beta_Lactam
cphA-01	GCGAGCTGCACAAGCTGAT	CGGCCCAGTCGCTCTTC	Beta_Lactam
cphA-02	GTGCTGATGGCGAGTTTCTG	GGTGTGGTAGTTGGTGTTGATCAC	Beta_Lactam
fox5	GGTTTGCCGCTGCAGTTC	GCGGCCAGGTGACCAA	Beta_Lactam
mecA	GGTTACGGACAAGGTGAAATACTGAT	TGTCTTTTAATAAGTGAGGTGCGTTAATA	Beta_Lactam
ndm-1	ATTAGCCGCTGCATTGAT	CATGTCGAGATAGGAAGTG	Beta_Lactam
pbp	CCGGTGCCATTGGTTTAGA	AAAATAGCCGCCCCAAGATT	Beta_Lactam
pbp2x	TTTCATAAGTATCTGGACATGGAAGAA	CCAAAGGAAACTTGCTTGAGATTAG	Beta_Lactam
Pbp5	GGCGAACTTCTAATTAATCCTATCCA	CGCCGATGACATTCTTCTTATCTT	Beta_Lactam
penA	AGACGGTAACGTATAACTTTTTGAAAGA	GCGTGTAGCCGGCAATG	Beta_Lactam
cmlA1-01	TAGGAAGCATCGGAACGTTGAT	CAGACCGAGCACGACTGTTG	Chloramphenicol
cmlA1-02	AGGAAGCATCGGAACGTTGA	ACAGACCGAGCACGACTGTTG	Chloramphenicol
cmx(A)	GCGATCGCCATCCTCTGT	TCGACACGGAGCCTTGGT	Chloramphenicol
cIntI-1(class1)	GGCATCCAAGCAGCAAG	AAGCAGACTTGACCTGA	Integron
intI-1(clinic)	CGAACGAGTGGCGGAGGGTG	TACCCGAGAGCTTGGCACCCA	Integron

carB	GGAGTGAGGCTGACCGTAGAAG	ATCGGCGAAACGCACAAA	MLSB
ereA	CCTGTGGTACGGAGAATTCATGT	ACCGCATTCGCTTTGCTT	MLSB
erm(34)	GCGCGTTGACGACGATTT	TGGTCATACTCGACGGCTAGAAC	MLSB
erm(35)	TTGAAAACGATGTTGCATTAAGTCA	TCTATAATCACAACTAACCACTTGAACGT	MLSB
erm(36)	GGCGGACCGACTTGCAT	TCTGCGTTGACGACGGTTAC	MLSB
ermA	TTGAGAAGGGATTTGCGAAAAG	ATATCCATCTCCACCATTAATAGTAAACC	MLSB
ermA/ermTR	ACATTTTACCAAGGAACTTGTGGAA	GTGGCATGACATAAACCTTCATCA	MLSB
ermB	TAAAGGGCATTTAACGACGAAACT	TTTATACCTCTGTTTGTTAGGGAATTGAA	MLSB
ermC	TTTGAAATCGGCTCAGGAAAA	ATGGTCTATTTCAATGGCAGTTACG	MLSB
ermF	CAGCTTTGGTTGAACATTTACGAA	AAATTCCTAAAATCACAACCGACAA	MLSB
ermJ/ermD	GGACTCGGCAATGGTCAGAA	CCCCGAAACGCAATATAATGTT	MLSB
ermK-01	GTTTGATATTGGCATTGTCAGAGAAA	ACCATTGCCGAGTCCACTTT	MLSB
ermK-02	GAGCCGCAAGCCCCTTT	GTGTTTCATTTGACGCGGAGTAA	MLSB
ermT-01	GTTCACTAGCACTATTTTTAATGACAGAAGT	GAAGGGTGTCTTTTTAATACAATTAACGA	MLSB
ermT-02	GTAAAATCCCTAGAGAATACTTTCATCCA	TGAGTGATATTTTTGAAGGGTGTCTT	MLSB
ermX	GCTCAGTGGTCCCCATGGT	ATCCCCCCGTCAACGTTT	MLSB
ermY	TTGTCTTTGAAAGTGAAGCAACAGT	TAACGCTAGAGAACGATTTGTATTGAG	MLSB
lmrA-01	TCGACGTGACCGTAGTGAACA	CGTGACTACCCAGGTGAGTTGA	MLSB
lnuA-01	TGACGCTCAACACACTCAAAAA	TTCATGCTTAAGTTCCATACGTGAA	MLSB
lnuB-01	TGAACATAATCCCCTCGTTTAAAGAT	TAATTGCCCTGTTTCATCGTAAATAA	MLSB
lnuB-02	AAAGGAGAAGGTGACCAATACTCTGA	GGAGCTACGTCAAACAACCAGTT	MLSB
lnuC	TGGTCAATATAACAGATGTAAACCAGATTT	CACCCCAGCCACCATCAA	MLSB
matA/mel	TAGTAGGCAAGCTCGGTGTTGA	CCTGTGCTATTTTAAGCCTTGTTTCT	MLSB
mdtA	CCTAACGGGCGTGACTTCA	TTCACCTGTTTCAAGGGTCAAA	MLSB
mefA	CCGTAGCATTGGAACAGCTTTT	AAACGGAGTATAAGAGTGCTGCAA	MLSB

mphA-01	CTGACGCGCTCCGTGTT	GGTGGTGCATGGCGATCT	MLSB
mphA-02	TGATGACCCTGCCATCGA	TTCGCGAGCCCCTCTTC	MLSB
mphB	CGCAGCGCTTGATCTTGTAG	TTACTGCATCCATACGCTGCTT	MLSB
mphC	CGTTTGAAGTACCGAATTGGAAA	GCTGCGGGTTTGCCTGTA	MLSB
msrA-01	CTGCTAACACAAGTACGATTCCAAAT	TCAAGTAAAGTTGTCTTACCTACACCATT	MLSB
msrC-01	TCAGACCGGATCGGTTGTC	CCTATTTTTTGGAGTCTTCTCTCTAATGTT	MLSB
oleC	CCCGGAGTCGATGTTCGA	GCCGAAGACGTACACGAACAG	MLSB
pikR1	TCGACATGCGTGACGAGATT	CCGCGAATTAGGCCAGAA	MLSB
pikR2	TCGTGGGCCAGGTGAAGA	TTCCCCTTGCCGGTGAA	MLSB
vatB-01	GGAAAAAGCAACTCCATCTCTTGA	TCCTGGCATAACAGTAACATTCTGA	MLSB
vatB-02	TTGGGAAAAAGCAACTCCATCT	CAATCCACACATCATTTCCAACA	MLSB
vatC-01	CGGAAATTGGGAACGATGTT	GCAATAATAGCCCCGTTTCCTA	MLSB
vatC-02	CGATGTTTGGATTGGACGAGAT	GCTGCAATAATAGCCCCGTTT	MLSB
vatE-01	GGTGCCATTATCGGAGCAAAT	TTGGATTGCCACCGACAAT	MLSB
vatE-02	GACCGTCCTACCAGGCGTAA	TTGGATTGCCACCGACAATT	MLSB
vgaA-01	CGAGTATTGTGGAAAGCAGCTAGTT	CCCGTACCGTTAGAGCCGATA	MLSB
vgaA-02	GACGGGTATTGTGGAAAGCAA	TTTCCTGTACCATTAGATCCGATAATT	MLSB
vgb-01	AGGGAGGGTATCCATGCAGAT	ACCAAATGCGCCCGTTT	MLSB
vgbB-01	CAGCCGGATTCTGGTCCTT	TACGATCTCCATTCAATTGGGTAAA	MLSB
vgbB-02	ATACGAGCTGCCTAATAAAGGATCTT	TGTGAACCACAGGGCATTATCA	MLSB
acrA-01	CAACGATCGGACGGGTTTC	TGGCGATGCCACCGTACT	Multidrug
acrA-02	GGTCTATCACCCTACGCGCTATC	GCGCGCACGAACATACC	Multidrug
acrA-03	CAGACCCGCATCGCATATT	CGACAATTTCGCGCTCATG	Multidrug
acrA-04	TACTTTGCGCGCCATCTTC	CGTGCGCGAACGAACAT	Multidrug

acrA-05	CGTGCGCGAACGAACA	ACTTTGCGCGCCATCTTC	Multidrug
acrB-01	AGTCGGTGTTCGCCGTTAAC	CAAGGAAACGAACGCAATACC	Multidrug
acrF	GCGGCCAGGCACAAAA	TACGCTCTTCCCACGGTTTC	Multidrug
acrR-01	GCGCTGGAGACACGACAAC	GCCTTGCTGCGAGAACAAA	Multidrug
acrR-02	GATGATACCCCCTGCTGTGAGA	ACCAAACAAGAAGCGCAAGAA	Multidrug
adeA	CAGTTCGAGCGCCTATTTCTG	CGCCCTGACCGACCAAT	Multidrug
ceoA	ATCAACACGGACCAGGACAAG	GGAAAGTCCGCTCACGATGA	Multidrug
cmeA	GCAGCAAAGAAGAAGCACCAA	AGCAGGGTAAGTAAAACTAAGTGGTAAATCT	Multidrug
cmr	CGGCATCGTCAGTGGAATT	CGGTTCCGAAAAAGATGGAA	Multidrug
emrD	CTCAGCAGTATGGTGGTAAGCATT	ACCAGGCGCCGAAGAAC	Multidrug
floR	ATTGTCTTCACGGTGTCCGTTA	CCGCGATGTCGTCGAACT	Multidrug
marR-01	GCGGCGTACTGGTGAAGCTA	TGCCCTGGTCGTTGATGA	Multidrug
mdetl1	ATACAGCAGTGGATATTGGTTTAATTGT	TGCATAAGGTGAATGTTCCATGA	Multidrug
mdtE/yhiU	CGTCGGCGCACTCGTT	TCCAGACGTTGTACGGTAACCA	Multidrug
mepA	ATCGGTCGCTCTTCGTTCAC	ATAAATAGGATCGAGCTGCTGGAT	Multidrug
mexA	AGGACAACGCTATGCAACGAA	CCGGAAAGGGCCGAAAT	Multidrug
mexD	TTGCCACTGGCTTTCATGAG	CACTGCGGAGAACTGTCTGTAGA	Multidrug
mexE	GGTCAGCACCGACAAGGTCTAC	AGCTCGACGTACTTGAGGAACAC	Multidrug
mexF	CCGCGAGAAGGCCAAGA	TTGAGTTCGGCGGTGATGA	Multidrug
mtrC-01	GGACGGGAAGATGGTCCAA	CGTAGCGTTCCGGTTCGAT	Multidrug
mtrC-02	CGGAGTCCATCGACCATTTG	ATCGTCGGCAAGGAGAATCA	Multidrug
mtrD-02	GGTCGGCACGCTCTTGTC	TGAAGAATTTGCGCACCACTAC	Multidrug
mtrD-03	CCGCCAAGCCGATATAGACA	GGCCGGGTTGCCAAA	Multidrug
oprD	ATGAAGTGGAGCGCCATTG	GGCCACGGCGAACTGA	Multidrug

oprJ	ACGAGAGTGGCGTCGACAA	AAGGCGATCTCGTTGAGGAA	Multidrug
pmrA	TTTGCAGGTTTTGTTCCTAATGC	GCAGAGCCTGATTTCTCCTTTG	Multidrug
putitive multidrug	AATTTTGCCGATTATTGCTGAAA	GATTGTCATCATTCGTTTATCACCAA	Multidrug
qac	CAATAATAACCGAAATAATAGGGACAAGTT	AATAAGTGTTCCTAGTGTTGGCCATAG	Multidrug
qacH-01	GTGGCAGCTATCGCTTGGAT	CCAACGAACGCCCACAA	Multidrug
qacH-02	CATCGTGCTTGTGGCAGCTA	TGAACGCCCAGAAGTCTAGTTTT	Multidrug
rarD-02	TGACGCATCGCGTGATCT	AAATTTTCTGTGGCGTCTGAATC	Multidrug
sdeB	CACTACCGCTTCCGCACTTAA	TGAAAAAACGGGAAAAGTCCAT	Multidrug
tolC-01	GGCCGAGAACCTGATGCA	AGACTTACGCAATTCCGGGTTA	Multidrug
tolC-02	CAGGCAGAGAACCTGATGCA	CGCAATTCCGGGTTGCT	Multidrug
tolC-03	GCCAGGCAGAGAACCTGATG	CGCAATTCCGGGTTGCT	Multidrug
ttgA	ACGCCAATGCCAAACGATT	GTCACGGCGCAGCTTGA	Multidrug
ttgB	TCGCCCTGGATGTACACCTT	ACCATTGCCGACATCAACAAC	Multidrug
yceE/mdtG-01	TGGCACAAAATATCTGGCAGTT	TTGTGTGGCGATAAGAGCATTAG	Multidrug
yceE/mdtG-02	TTATCTGTTTTCTGCTCACCTTCTTTT	GCGTGGTGACAAACAGGCTTA	Multidrug
yceL/mdtH-01	TCGGGATGGTGGGCAAT	CGATAACCGAGCCGATGTAGA	Multidrug
yceL/mdtH-02	CGCGTGAAACCTTAAGTGCTT	AGACGGCTAAACCCCATATAGCT	Multidrug
yceL/mdtH-03	CTGCCGTTAAATGGATGTATGC	ACTCCAGCGGGCGATAGG	Multidrug
yidY/mdtL-01	GCAGTTGCATATCGCCTTCTC	CTTCCCGGCAAACAGCAT	Multidrug
yidY/mdtL-02	TGCTGATCGGGATTCTGATTG	CAGGCGCGACGAACATAAT	Multidrug
dfrA1	GGAATGGCCCTGATATTCCA	AGTCTTGCGTCCAACCAACAG	Sulfonamide
dfrA12	CCTCTACCGAACCGTCACACA	GCGACAGCGTTGAAACAACTAC	Sulfonamide
folA	CGAGCAGTTCCTGCCAAAG	CCCAGTCATCCGGTTCATAATC	Sulfonamide
sul1	CAGCGCTATGCGCTCAAG	ATCCCGCTGCGCTGAGT	Sulfonamide
sul2	TCATCTGCCAAACTCGTCGTTA	GTCAAAGAACGCCGCAATGT	Sulfonamide

sulA/folP-01	CAGGCTCGTAAATTGATAGCAGAAG	CTTTCCTTGCGAATCGCTTT	Sulfonamide
sulA/folP-03	CACGGCTTCGGCTCATGT	TGCCATCCTGTGACTAGCTACGT	Sulfonamide
tet(32)	CCATTACTTCGGACAACGGTAGA	CAATCTCTGTGAGGGCATTTAACA	Tetracycline
tet(34)	CTTAGCGCAAACAGCAATCAGT	CGGTGATACAGCGCGTAAACT	Tetracycline
tet(35)	ACCCCATGACGTACCTGTAGAGA	CAACCCACACTGGCTACCAGTT	Tetracycline
tet(36)-01	AGAATACTCAGCAGAGGTCAGTTCCT	TGGTAGGTCGATAACCCGAAAAT	Tetracycline
tet(36)-02	TGCAGGAAAGACCTCCATTACAG	CTTTGTCCACACTTCCACGTACTATG	Tetracycline
tet(37)	GAGAACGTTGAAAAGGTGGTGAA	AACCAAGCCTGGATCAGTCTCA	Tetracycline
tetA-01	GCTGTTTGTTCTGCCGGAAA	GGTTAAGTTCCTTGAACGCAAACT	Tetracycline
tetA-02	CTCACCAGCCTGACCTCGAT	CACGTTGTTATAGAAGCCGCATAG	Tetracycline
tetB-01	AGTGCGCTTTGGATGCTGTA	AGCCCCAGTAGCTCCTGTGA	Tetracycline
tetB-02	GCCCAGTGCTGTTGTTGTCAT	TGAAAGCAAACGGCCTAAATACA	Tetracycline
tetC-01	CATATCGCAATACATGCGAAAAA	AAAGCCGCGGTAAATAGCAA	Tetracycline
tetC-02	ACTGGTAAGGTAAACGCCATTGTC	ATGCATAAACCAGCCATTGAGTAAG	Tetracycline
tetD-01	TGCCGCGTTTGATTACACA	CACCAGTGATCCCGGAGATAA	Tetracycline
tetD-02	TGTCATCGCGCTGGTGATT	CATCCGCTTCCGGGAGAT	Tetracycline
tetE	TTGGCGCTGTATGCAATGAT	CGACGACCTATGCGATCTGA	Tetracycline
tetG-01	TCAACCATTGCCGATTCGA	TGGCCCGGCAATCATG	Tetracycline
tetG-02	CATCAGCGCCGGTCTTATG	CCCCATGTAGCCGAACCA	Tetracycline
tetH	TTTGGGTCATCTTACCAGCATTAA	TTGCGCATTATCATCGACAGA	Tetracycline
tetJ	GGGTGCCGCATTAGATTACCT	TCGTCCAATGTAGAGCATCCATA	Tetracycline
tetK	CAGCAGTCATTGGAAAATTATCTGATTATA	CCTTGTACTAACCTACCAAAAATCAAAATA	Tetracycline
tetL-01	AGCCCGATTTATTCAAGGAATTG	CAAATGCTTTCCCCCTGTTCT	Tetracycline
tetL-02	ATGGTTGTAGTTGCGCGCTATAT	ATCGCTGGACCGACTCCTT	Tetracycline
tetM-01	CATCATAGACACGCCAGGACATAT	CGCCATCTTTTGCAGAAATCA	Tetracycline

tetM-02	TAATATTGGAGTTTTAGCTCATGTTGATG	CCTCTCTGACGTTCTAAAAGCGTATTAT	Tetracycline
tetO-01	ATGTGGATACTACAACGCATGAGATT	TGCCTCCACATGATATTTTTCCT	Tetracycline
tetPA	AGTTGCAGATGTGTATAGTCGTAAACTATCTATT	TGCTACAAGTACGAAAACAAAACTAGAA	Tetracycline
tetPB-01	ACACCTGGACACGCTGATTTT	ACCGTCTAGAACGCGGAATG	Tetracycline
tetPB-02	TGATACACCTGGACACGCTGAT	CGTCCAAAACGCGGAATG	Tetracycline
tetPB-03	TGGGCGACAGTAGGCTTAGAA	TGACCCTACTGAAACATTAGAAATATACCT	Tetracycline
tetPB-04	AGTGGTGCAAATACTGAAAAAGTTGT	TTTGTTCCTTCGTTTTGGACAGA	Tetracycline
tetPB-05	CTGAAGTGGAGCGATCATTCC	CCCTCAACGGCAGAAATAACTAA	Tetracycline
tetQ	CGCCTCAGAAGTAAGTTCATACACTAAG	TCGTTCATGCGGATATTATCAGAAT	Tetracycline
tetR-02	CGCGATAGACGCCTTCGA	TCCTGACAACGAGCCTCCTT	Tetracycline
tetR-03	CGCGATGGAGCAAAAGTACAT	AGTGAAAAACCTTGTTGGCATAAAA	Tetracycline
tetS	TTAAGGACAAACTTTCTGACGACATC	TGTCTCCCATTGTTCTGGTTCA	Tetracycline
tetT	CCATATAGAGGTTCCACCAAATCC	TGACCCTATTGGTAGTGGTTCTATTG	Tetracycline
tetU-01	GTGGCAAAGCAACGGATTG	TGCGGGCTTGCAAAACTATC	Tetracycline
tetV	GCGGGAACGACGATGTATATC	CCGCTATCTCACGACCATGAT	Tetracycline
tetX	AAATTTGTTACCGACACGGAAGTT	CATAGCTGAAAAAATCCAGGACAGTT	Tetracycline
IS613	AGGTTCGGACTCAATGCAACA	TTCAGCACATACCGCCTTGAT	Transposase
tnpA-01	CATCATCGGACGGACAGAATT	GTCGGAGATGTGGGTGTAGAAAGT	Transposase
tnpA-02	GGGCGGGTCGATTGAAA	GTGGGCGGATCTGCTT	Transposase
tnpA-03	AATTGATGCGGACGGCTTAA	TCACCAAACTGTTTATGGAGTCGTT	Transposase
tnpA-04	CCGATCACGGAAAGCTCAAG	GGCTCGCATGACTTCGAATC	Transposase
tnpA-05	GCCGCACTGTCGATTTTTATC	GCGGGATCTGCCACTTCTT	Transposase
tnpA-07	GAAACCGATGCTACAATATCCAATTT	CAGCACCGTTTGCAGTGTAAG	Transposase
Tp614	GGAAATCAACGGCATCCAGTT	CATCCATGCGCTTTTGTCTCT	Transposase
vanA	AAAAGGCTCTGAAAACGCAGTTAT	CGGCCGTTATCTTGTAAAAACAT	Vancomycin

vanB-01	TTGTCGGCGAAGTGGATCA	AGCCTTTTTCCGGCTCGTT	Vancomycin
vanB-02	CCGGTCGAGGAACGAAATC	TCCTCCTGCAAAAAAAGATCAAC	Vancomycin
vanC-01	ACAGGGATTGGCTATGAACCAT	TGACTGGCGATGATTTGACTATG	Vancomycin
vanC-03	AAATCAATACTATGCCGGGCTTT	CCGACCGCTGCCATCA	Vancomycin
vanC1	AGGCGATAGCGGGTATTGAA	CAATCGTCAATTGCTCATTTCC	Vancomycin
vanC2/vanC3	TTTGACTGTCGGTGCTTGTGA	TCAATCGTTTCAGGCAATGG	Vancomycin
vanG	ATTTGAATTGGCAGGTATACAGGTTA	TGATTTGTCTTTGTCCATACATAATGC	Vancomycin
vanHB	GAGGTTTCCGAGGCGACAA	CTCTCGGCGGCAGTCGTAT	Vancomycin
vanHD	GTGGCCGATTATACCGTCATG	CGCAGGTCATTCAGGCAAT	Vancomycin
vanRA-01	CCCTTACTCCCACCGAGTTTT	TTCGTCGCCCCATATCTCAT	Vancomycin
vanRA-02	CCACTCCGGCCTTGTCATT	GCTAACCACATTCCCCTTGTTTT	Vancomycin
vanRB	GCCCTGTCGGATGACGAA	TTACATAGTCGTCTGCCTCTGCAT	Vancomycin
vanRC	TGCGGGAAAAACTGAACGA	CCCCCCATACGGTTTTGATTA	Vancomycin
vanRC4	AGTGCTTTGGCTTATCTCGAAAA	TCCGGCAGCATCACATCTAA	Vancomycin
vanRD	TTATAATGGCAAGGATGCACTAAAGT	CGTCTACATCCGGAAGCATGA	Vancomycin
vanSA	CGCGTCATGCTTTCAAAATTC	TCCGCAGAAAGCTCAATTTGTT	Vancomycin
vanSB	GCGCGGCAAATGACAAC	TTTGCCATTTTATTCGCACTGT	Vancomycin
vanSC-02	GCCATCAGCGAGTCTGATGA	CAGCTGGGATCGTTTTTCCTT	Vancomycin
vanSE	TGGCCGAAGAAGCAGGAA	CAATAATACTCGTCAAAGGAGTTCTCA	Vancomycin
vanTC-01	CACACGCATTTTTTCCCATCTAG	CAGCCAACAGATCATCAAAACAA	Vancomycin
vanTC-02	ACAGTTGCCGCTGGTGAAG	CGTGGCTGGTCGATCAAAA	Vancomycin
vanTE	GTGGTGCCAAGGAAGTTGCT	CGTAGCCACCGCAAAAAAAT	Vancomycin
vanTG	CGTGTAGCCGTTCCGTTCTT	CGGCATTACAGGTATATCTGGAAA	Vancomycin
vanWB	CGGACAAAGATACCCCCTATAAAG	AAATAGTAAATTGCTCATCTGGCACAT	Vancomycin
vanWG	ACATTTTCATTTTGGCAGCTTGTAC	CCGCCATAAGAGCCTACAATCT	Vancomycin

vanXA	CGCTAAATATGCCACTTGGGATA	TCAAAAGCGATTCAGCCAACT	Vancomycin
vanXB	AGGCACAAAATCGAAGATGCTT	GGGTATGGCTCATCAATCAACTT	Vancomycin
vanXD	TAAACCGTGTTATGGGAACGAA	GCGATAGCCGTCCCATAAGA	Vancomycin
vanYB	GGCTAAAGCGGAAGCAGAAA	GATATCCACAGCAAGACCAAGCT	Vancomycin
vanYD-01	AAGGCGATACCCTGACTGTCA	ATTGCCGGACGGAAGCA	Vancomycin
vanYD-02	CAAACGGAAGAGAGGTCACTTACA	CGGACGGTAATAGGGACTGTTC	Vancomycin
bacA-01	CGGCTTCGTGACCTCGTT	ACAATGCGATACCAGGCAAAT	Others
bacA-02	TTCCACGACACGATTAAGTCATTG	CGGCTCTTTCGGCTTCAG	Others
catA1	GGGTGAGTTTCACCAGTTTTGATT	CACCTTGTCGCCTTGCGTATA	Others
catB3	GCACTCGATGCCTTCCAAAA	AGAGCCGATCCAAACGTCAT	Others
catB8	CACTCGACGCCTTCCAAAG	CCGAGCCTATCCAGACATCATT	Others
cfr	GCAAAATTCAGAGCAAGTTACGAA	AAAATGACTCCCAACCTGCTTTAT	Others
ereB	GCTTTATTTCAGGAGGCGGAAT	TTTTAAATGCCACAGCACAGAATC	Others
fabK	TTTCAGCTCAGCACTTTGGTCAT	AAGGCATCTTTTTCAGCCAGTTC	Others
fosB	TCACTGTAACTAATGAAGCATTAGACCAT	CCATCTGGATCTGTAAAGTAAAGAGATC	Others
fosX	GATTAAGCCATATCACTTTAATTGTGAAAG	TCTCCTTCCATAATGCAAATCCA	Others
imiR	CCGGACTAGAGCTTCATGTAAGC	CCCACGCGGTACTCTTGTAAA	Others
nimE	TGCGCCAAGATAGGGCATA	GTCGTGAATTCGGCAGGTTTA	Others
nisB	GGGAGAGTTGCCGATGTTGTA	AGCCACTCGTTAAAGGGCAAT	Others
pncA	GCAATCGAGGCGGTGTTC	TTGCCGCAGCCAATTCA	Others
qacA	TGGCAATAGGAGCTATGGTGTTT	AAGGTAACACTATTTTCGGTCCAAATC	Others
qacA/qacB	TTTAGGCAGCCTCGCTTCA	CCGAATCCAAATAAAACCCAATAA	Others
qacEdelta1-01	TCGCAACATCCGCATTAAAA	ATGGATTTCAGAACCAGAGAAAGAAA	Others
qacEdelta1-02	CCCCTTCCGCCGTTGT	CGACCAGACTGCATAAGCAACA	Others
qnrA	AGGATTTCTCACGCCAGGATT	CCGCTTTCAATGAAACTGCAA	Others

sat4	GAATGGGCAAAGCATAAAAACTTG	CCGATTTTGAAACCACAATTATGATA	Others
speA	GCAAGAGGTATTTGCTCAACAAGA	CAGGGTCACCCTCATAAAGAAAA	Others
